# Spread and seasonality of COVID-19 pandemic confirmed cases in sub-Saharan Africa: experience from Democratic Republic of Congo, Nigeria, Senegal, and Uganda

**DOI:** 10.1186/s12879-023-08168-1

**Published:** 2023-03-29

**Authors:** Ayo S. Adebowale, Rotimi F. Afolabi, Segun Bello, Mobolaji M. Salawu, Eniola A. Bamgboye, Ikeola Adeoye, Magbagbeola D. Dairo, Betty Kivumbi, Irene Wanyana, Ibrahima Seck, Issakha Diallo, Mamadou M. M. Leye, Oumar Bassoum, Mane Fall, Rawlance Ndejjo, Steven N. Kabwama, Mala Ali Mapatano, Marc Bosonkie, Landry Egbende, Alice Namale, Susan Kizito, Rhoda K. Wanyenze, Olufunmilayo I. Fawole

**Affiliations:** 1 Department of Epidemiology and Medical Statistics, Faculty of Public Health, College of Medicine, University of Ibadan, Ibadan, Nigeria; 2 Population and Health Research Entity, School of Social Sciences, North-West University, Mafikeng, South Africa; 3 Department of Mathematics, School of Physical Sciences, College of Natural Sciences, Makerere University, Kampala, Uganda; 4 Department of Epidemiology and Biostatistics, School of Public Health, College of Health Sciences, Makerere University, Kampala, Uganda; 5 Department of Preventive Medicine and Public Health, University Cheikh Antar Diop, Dakar, Senegal; 6 Department of Disease Control and Environmental Health, School of Public Health, College of Health Sciences, Makerere University, Kampala, Uganda; 7 Department of Community Health and Behavioral Sciences, School of Public Health, College of Health Sciences, Makerere University, Kampala, Uganda; 8 Department of Nutrition, School of Public Health, University of Kinshasa, Kinshasa, Democratic Republic of Congo; 9 School of Public Health, Makerere University, Kampala, Uganda

**Keywords:** COVID-19, Trigonometric model, Mathematical projection, Sub-Saharan Africa

## Abstract

**Background:**

The COVID-19 pandemic has impacted the world negatively with huge health and socioeconomic consequences. This study estimated the seasonality, trajectory, and projection of COVID-19 cases to understand the dynamics of the disease spread and inform response interventions.

**Method:**

Descriptive analysis of daily confirmed COVID-19 cases from January 2020 to 12^th^ March 2022 was conducted in four purposefully selected sub-Saharan African countries (Nigeria, Democratic Republic of Congo (DRC), Senegal, and Uganda). We extrapolated the COVID-19 data from (2020 to 2022) to 2023 using a trigonometric time series model. A decomposition time series method was used to examine the seasonality in the data.

**Results:**

Nigeria had the highest rate of spread (β) of COVID-19 (β = 381.2) while DRC had the least rate (β = 119.4). DRC, Uganda, and Senegal had a similar pattern of COVID-19 spread from the onset through December 2020. The average doubling time in COVID-19 case count was highest in Uganda (148 days) and least in Nigeria (83 days). A seasonal variation was found in the COVID-19 data for all four countries but the timing of the cases showed some variations across countries. More cases are expected in the 1^st^ (January-March) and 3^rd^ (July–September) quarters of the year in Nigeria and Senegal, and in the 2^nd^ (April-June) and 3^rd^ (October-December) quarters in DRC and Uganda.

**Conclusion:**

Our findings show a seasonality that may warrant consideration for COVID-19 periodic interventions in the peak seasons in the preparedness and response strategies.

## Background

The SARS-CoV-2 infection, which causes the COVID-19 disease continues to be a public health concern since the outbreak of the disease was recorded in late 2019. The disease has affected almost every country around the world and as of 7^th^ February 2022, close to 400 million cases had been reported with over 5.5 million deaths [1]. The Covid-19 case fatality rate of 0.015% is one of the highest among emerging and re-emerging diseases globally [1, 2]. The three countries with the highest burden of disease are found in the North-America, Europe, and Asia. Amongst other countries that recorded at least a case of the disease, the United States of America has the highest number of cases (78,017,402) with 926,029 deaths and 2,772 deaths per 1,000,000 population followed by India with 42,272,014 cases, Brazil and France, respectively. South Africa, was the most affected country in Africa with 3,623,962 confirmed cases and 1,584 deaths per 1,000,000 population [1]. Some African countries like Morocco, Tunisia, Ethiopia, Libya, and Egypt also recorded cases of above 400,000 [1, 2].

Despite the extensive spread of COVID-19, mortality was greatly concentrated in developed countries. Developing countries which account for 85% of the world population, had about 20% of the global COVID–19-related deaths [2, 3]. Variations in atmospheric conditions, demographic profile, socioeconomic status, surveillance system, and policy responses possibly account for this difference [4]. Pre-existing medical conditions such as cardiovascular disease, diabetes, chronic respiratory disease, hypertension, and cancer, among others increase the risk of dying due to COVID-19 [3, 5]. Although the severity of deaths that are attributable to COVID-19 is higher in North America, Europe, and Asia than in the most affected sub-Saharan African countries, the socioeconomic consequences and health impact of the disease were enormous in the region [6]. Therefore, it is pertinent to conduct a critical appraisal of the trajectory of the COVID-19 pandemic in sub-Saharan Africa. In the current study, the dynamics of COVID-19 were examined in four purposefully chosen countries – the Democratic Republic of Congo, Nigeria, Senegal, and Uganda, using the trigonometric regression model.

The trigonometrical ratio method has been used in the past for modeling the dynamics of infectious diseases. Several studies have used trigonometric regression in the analysis of public health surveillance data to examine the seasonality and dynamics of other infectious diseases, including multiple sclerosis relapse, among others [7–9]. Central to the success of the previous studies that used this modeling approach was its flexibility in terms of description, prediction, and consistency in patterns like the annual cycle of peaks and troughs usually experienced in biological phenomena that have seasonality tendencies. Analysis of seasonality, the growth patterns of the disease, and periodic forecasting at different points in a year can guide planning, preparedness, and prioritization of resources to alleviate the disease spread during the period. This model has not been fully utilized to guide the COVID-19 response. In this study, trigonometrical regression was used to model the pattern of COVID-19 cases in Nigeria, the Congo Democratic Republic, Senegal, and Uganda.

The seasonality of flu-like COVID-19 in tropical countries can be attributed to; mobility to low/high-risk locations, meteorological drivers, seasonal allergens, overcrowding, poor living arrangements, and population susceptibility. Other factors that can drive seasonal patterns in the dynamics of infectious diseases include periodic variations in; human activity and behavior, the human immune system, vitamin D levels, melatonin, and pathogen infectivity [10]. While environmental factors can impact the abundance of pathogens through housing ventilation and outdoor activity in natural ultraviolet light, adequate vitamin D may be needed during the cold season to decrease infection rates [10]. During the rainy season, flu spreads more easily [10]. Therefore, to what extent is COVID-19 spreading faster during the rainy season in the studied tropical African countries? This primary question about the seasonality of COVID-19 remains unresolved, particularly in tropical regions, where little is known about the seasonality of the disease.

Previous study has established that there was a similarity in the incidence of the community outbreak and seasonal patterns of COVID-19 and other Influenza-Like Illnesses (ILI) [11]. The first and second wave of COVID-19 also suggests seasonality, analogous to the Influenza-Like Illnesses (ILI) seasonality [12]. These assertions are based on mere speculations but are not evidence-based. The seasonality of COVID-19 has been confirmed, especially in the temperate climate zone countries [13]. Therefore this study, intending to establish the seasonality of COVID-19 in tropical and sub-tropical climate zones as well, is of great interest. Therefore, time series analysis was used to examine the seasonality of COVID-19 in each of the studied countries. In addition, the pattern of COVID-19 was modeled and the COVID-19 doubling time was examined from January 2020 to 12^th^ March 2022. Identification of the seasonality of COVID-19 may offer the possibilities for preventive strategies and can promote the development of effective policies, and provides an opportunity for the efficient and effective allocation and use of resources particularly in a poor resource setting like Nigeria.

## Methods

### Study area

Four countries were selected for this study; the Democratic Republic of Congo, Nigeria, Senegal, and Uganda. The countries had coordination structures that permitted multi-sectorial engagement in the COVID-19 response including non-governmental organizations and international agencies like WHO. In Nigeria, a Presidential COVID-19 Taskforce was constituted than comprised ministries and government agencies, and an inter-ministerial multi-sectoral technical working group with a secretariat at the Federal Ministry of Health. The DRC had a multisectoral response committee (Comite multisectoral de reposte) with a secretariat at the Ministry of Health. Senegal had a National Epidemic Management Committee (CNGE) that allowed for multi-sectoral engagement. Uganda had a National Taskforce that included various ministries as well as a strategic-level committee. Additionally, the countries were included in this study because of their historical experience in managing public health emergencies of international concern [14–17].

**Fig. 1:**
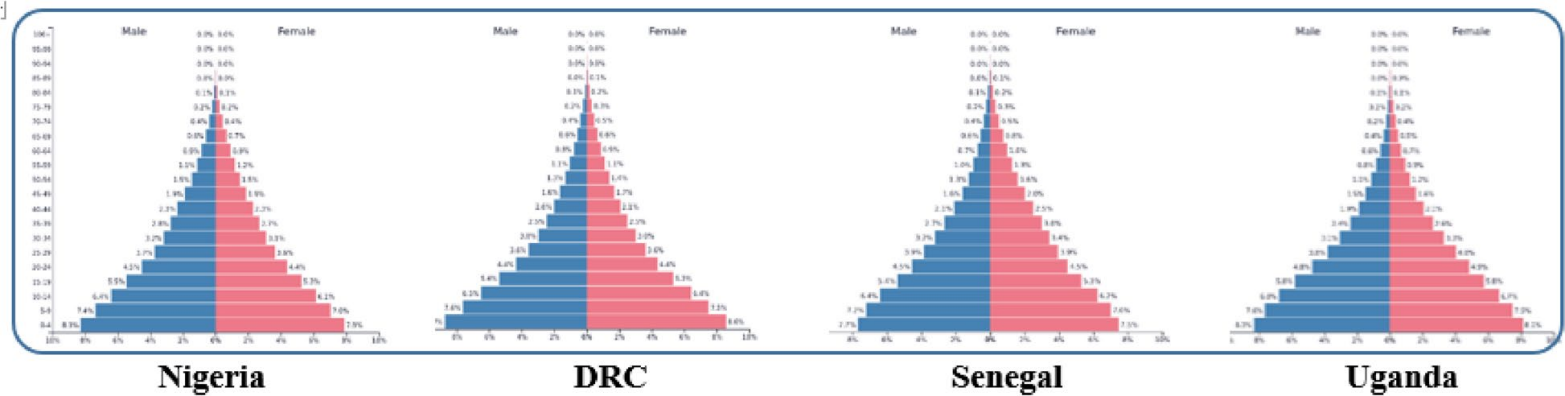
Population pyramid of Nigeria, DRC, Senegal, and Uganda. Source: https://www.populationpyramid.net/uganda/2022/

While Nigeria and Senegal are countries from West Africa, the Democratic Republic of Congo and Uganda are from Central and East Africa respectively. The countries have a similar age structure with a predominantly young population (Fig. 1) and are classified as low-income according to World Bank. Nigeria has the highest population (215,746,933), followed by the Democratic Republic of Congo (95,240,781), Uganda (48,432,873), and Senegal (17,653,669). Life expectancy was 55.75 in Nigeria, 61.60 in the Democratic Republic of Congo, 64.38 in Uganda, and 68.87 in Senegal [18]. The countries are among the tropical countries in sub-Saharan Africa and they have hot, wet, and humid weather. They have a tropical climate, in which the average monthly temperatures are about 18 °C or higher and the year consists of two seasons: the wet/rainy season, in which most rainfall occurs, and the dry season [19].

The first case of COVID-19 was confirmed on the 27^th^ of February, 2020 in Nigeria, the 2^nd^ of March, 2020 in Senegal, the 10^th^ of March, 2020 in the Democratic Republic of Congo (DRC), and the 21^st^ of March in Uganda [1]. Data on basic COVID-19 indices and population density are contained in Table 1 [1, 20].

**Table 1:** Basic COVID-19 indices in Nigeria, DRC, Senegal and Uganda

**Country**	**Population Density/km**^**2**^	**Total Cases**	**Total Deaths**	**CFR**	**Total Cases/IM Population**	**Deaths/1 M Population**
**Nigeria**	246	254,953	3,142	0.0123	1,187	15
**DRC**	18	86,315	1,335	0.0155	917	14
**Senegal**	92	85,806	1,964	0.0229	4906	112
**Uganda**	242	163,517	3,594	0.0220	3394	75

Sources: https://www.worldometers.info/coronavirus/ & https://worldpopulationreview.com/: Data accessed on 12^th^ March, 2022

The countries demonstrated variation in their COVID-19 responses, both in terms of the scope and intensity of non-pharmaceutical interventions and in their outcomes; they had historical experience in managing epidemics of global concern, such as yellow fever, Ebola virus disease, and Marburg virus disease [21–23]; there were existing partnerships between local research institutions and government offices which eased access to COVID-19 and other health systems data and enabled the translation of research findings to evidence-based policy and practice; and the mixture of Francophone (the DRC and Senegal) and Anglophone (Nigeria and Uganda) countries enhances South-to-South cross-learning networks and communities of practice. The research was led by partners at the Makerere University School of Public Health in collaboration with the University of Kinshasa, Université Cheikh Anta Diop, and the University of Ibadan.

### Data source and extraction

The data on the daily confirmed COVID-19 cases up to 12^th^ March 2022 were extracted from the data repository of the Worldometer [1] and European Centre for Disease Prevention and Control [2]. The data were aggregated on monthly basis for those months where complete data were available and aggregated quarterly afterward. The quarterly classifications were: Quarter 1: January–March, Quarter 2: April–June, Quarter 3: July–September, and Quarter 4: November–December.

### Data analysis

Microsoft Excel and SPSS packages were used for data analysis. Charts and line graphs were used to present the data for each of the countries. The COVID-19 data were available for 2 years and as such not suitable to examine the quarterly trend and seasonal variation in COVID-19 cases which requires a minimum of 3 years. To ensure that the data span through a period of at least 3 years, a trigonometrical time series model was used to extrapolate the data to 2023 with the assumption that the present trend in COVID-19 daily confirmed cases is sustained throughout the period. The processes involved in the projection and time series modeling are itemized below.

#### Time series

The COVID-19 data was plotted for the preliminary examination of the trend for each country. Auto correlations were calculated and used to examine the existence of serial correlation in the series. Correlogram was also plotted as a preliminary check to ascertain whether the series had a seasonal variation of the trigonometrical type or not. A periodogram and spectra density was plotted afterward. The periodogram was used to ascertain the dominant frequencies or cyclical behavior in the COVID-19 cases observed over a period of time. The periodogram graphs measure the relative importance of the exact frequency values that explain the pattern of oscillation exhibited in the observed COVID-19 cases. The spectra analysis reveals the periods or finite time intervals where COVID-19 data are concentrated. In addition, the spectral analysis was used to view the observed COVID-19 cases as a sum of cosine waves with varying amplitudes and frequencies. To assess the seasonality in the data, the data were aggregated in 3 months to represent four quarters in a year. Thereafter, a regression model was used to fit a trigonometric curve to the data. Seasonality occurs when data experiences regular and predictable fluctuations that persist at a particular period in every calendar year. Seasonality in the pattern and growth of an infectious disease might be due to weather and the occurrence of activities that promote interactions of the population in a specific period of a year. It provides a clear picture of the yearly spread of the disease.

#### Building trigonometrical model

The trigonometrical model was used to smooth the available data beyond 2 year. Trigonometric functions are periodic and tie together time points one cycle apart. It has the advantage of smoothening the irregularities in the data through the imposition of a certain continuity between neighboring points [7–9]. The plot of daily observed COVID-19 cases shows that the data repeats itself at an interval of time but is not fixed and may be considered a a periodic pattern and as such this may suggest a sinusoidal function of the form. The amplitude of a sinusoidal function is the distance from either the average value to the maximum value or from the average value to the minimum value. The functions oscillate above and below the average value, are periodic, and repeat values in set cycles.

1 $${\mathrm{Y}}_{\mathrm{t}}=\mathrm{\upmu} +\mathrm{acos}\left(\mathrm{\upomega} (\mathrm{t}+\mathrm{k})+\mathrm{\uptheta} \right)+\mathrm{\upvarepsilon}_{\mathrm{t}}$$

where: μ = mean number of COVID-19 cases per month. Y_t_ = the number of COVID-19 cases occurring in the t-th ordinal month starting from the month when the outbreak occurs in each country, ω = frequency of the periodic variation = 2πf (since f = 1/T and T = 12 months), t = Time period (in a month), a = Amplitude of the data, θ = phase which locates the peak, ε_t_ = Error or residual term, and k = Adjustment factor used to fit the data. The parameter (ωt + θ) is measured in radians. Through re-parameterization, Eq. 1 was transformed into a linear multiple regression of the form.

2 $${\mathrm{Y}}_{\mathrm{t}}=\upmu +{\mathrm{\alpha}}_{1}{\mathrm{q}}_{1\mathrm{t}}+{\mathrm{\alpha}}_{2}{\mathrm{q}}_{2\mathrm{t}}+{\upvarepsilon}_{\mathrm{t}}$$

where; $${\mathrm{\alpha }}_{1}=\mathrm{acos\theta }; {\mathrm{\alpha }}_{2}=-\mathrm{asin\theta }; {\mathrm{q}}_{1\mathrm{t}}=\mathrm{cos\omega t}; {\mathrm{q}}_{2\mathrm{t}}=\mathrm{sin\omega t};$$

$$\mathrm{a}=\sqrt{{\mathrm{\alpha }}_{1}^{2}+{\mathrm{\alpha }}_{2}^{2}};\uptheta ={\mathrm{tan}}^{-1}\left(-\frac{{\mathrm{\alpha }}_{1}}{{\mathrm{\alpha }}_{2}}\right).$$

$$\mathrm{\mu \,is \,estimated \,by \,X}=\frac{1}{\mathrm{N}}{\sum }_{\mathrm{t}=1}^{\mathrm{N}}{\mathrm{X}}_{\mathrm{t}}$$

$${\mathrm{\alpha }}_{1}=\frac{2}{\mathrm{N}}{\sum }_{\mathrm{t}=1}^{\mathrm{N}}{\mathrm{X}}_{\mathrm{t}}\mathrm{cos\omega t}; {\mathrm{\alpha }}_{2}=\frac{2}{\mathrm{N}}{\sum }_{\mathrm{t}=1}^{\mathrm{N}}{\mathrm{X}}_{\mathrm{t}}\mathrm{sin\omega t};$$

The justification that is in the introduction can be moved and brought here.

#### Decomposition of the time series model

Decomposition models are useful when the parameters describing a time series are not changing over time which is the case for the trigonometrical model built for the data for each country. This is with the view to separating the time series into several factors: trend, seasonal, cyclical, and irregular (error). The plot of the smoothed data for each country follows an additive time series model. This is because the parameters describing the series are not changing over time with the assumption that the variation in the trend is constant over a period of time [24]. The model is as follows:

3 $${\mathrm{O}}_{\mathrm{t}}={\mathrm{T}}_{\mathrm{t}}+{\mathrm{S}}_{\mathrm{t}}+{\mathrm{C}}_{\mathrm{t}}+{\mathrm{I}}_{\mathrm{t}}$$

O_t_ = the observed quarterly COVID-19 data in time t; T_t_ = the trend component in time t; S_t_ = the seasonal component in time t; C_t_ = the cyclical component in time t; I_t_ = the irregular component in time t. The quarterly pattern of the smoothed COVID-19 cases in each of the studied countries has no indication of C_t_ and I_t_. Therefore, C_t_ = 0 and I_t_ = 0, Eq. (3) is then transformed to ${\mathrm{S}}_{\mathrm{t}}={\mathrm{O}}_{\mathrm{t}}-{\mathrm{T}}_{\mathrm{t}}$

The smoothed monthly confirmed cases of COVID-19 were aggregated for each quarter of the year (${\mathrm{O}}_{\mathrm{t}}$). A 3-point moving average method was used to determine the trend line (${\mathrm{T}}_{\mathrm{t}}$) for the quarterly data. The moving average method was used to eliminate seasonal variations and irregular fluctuations from the data. The trend line was deseasonalized using ${\mathrm{S}}_{\mathrm{t}}={\mathrm{O}}_{\mathrm{t}}-{\mathrm{T}}_{\mathrm{t}}$ to determine the seasonal index and this was adjusted afterward.

4 $$\mathrm{Average \,Seasonal \,Variation \,per \,Quarter}=\frac{\sum_{\mathrm{i},\mathrm{j}}{\mathrm{S}}_{{\mathrm{t}}_{\mathrm{ij}}}}{L}$$

5 $$\mathrm{Adjustment \,factor }\,(\mathrm{AF})=\sum\nolimits_{j=1}^{r}\left(\frac{\sum_{\mathrm{i},\mathrm{j}}{\mathrm{S}}_{{\mathrm{t}}_{\mathrm{ij}}}}{L}\right)$$

6 $$\mathrm{Adjusted \,Seasonal \,Index }\left(\mathrm{ASI}\right)=\frac{\sum_{\mathrm{i},\mathrm{j}}{\mathrm{S}}_{{\mathrm{t}}_{\mathrm{ij}}}}{L}-\sum\nolimits_{j=1}^{r}\left(\frac{\sum_{\mathrm{i},\mathrm{j}}{\mathrm{S}}_{{\mathrm{t}}_{\mathrm{ij}}}}{L}\right)$$

$$Year \,i=\mathrm{1,2},\mathrm{3,4}; Quarter \,j=\mathrm{1,2},\mathrm{3,4}$$

where; L is the number of years where the S_t_ is available, and r is 4, the number of quarters in a year. The adjusted seasonal index (ASI) was used to assess the seasonality of COVID-19 in each country. A quarter with a higher ASI value is expected to experience high cases of COVID-19.

## Results

**Fig. 2:**
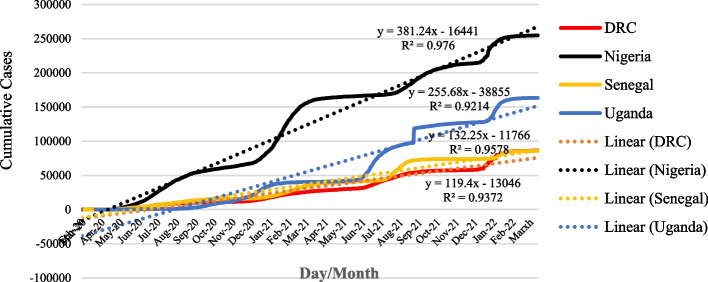
Cumulative daily Cases of COVID-19 in DRC, Nigeria, Senegal and Uganda

The cumulative daily cases of COVID-19 as of 12^th^ March 2022 in each of the countries are presented in Fig. 2. The number of confirmed cases and the rate of spread (β) of the disease were highest in Nigeria (β =  + 381.24), followed by Uganda (β =  + 255.68), Senegal (β =  + 132.25), and DRC (β =  + 119.4). The data show a similar pattern of the spread of COVID-19 in DRC, Uganda, and Senegal from the onset of the disease through December 2020. While this pattern was sustained in DRC and Senegal till 12^th^ March 2022, deviation from the pattern was experienced in Uganda.

**Fig. 3:**
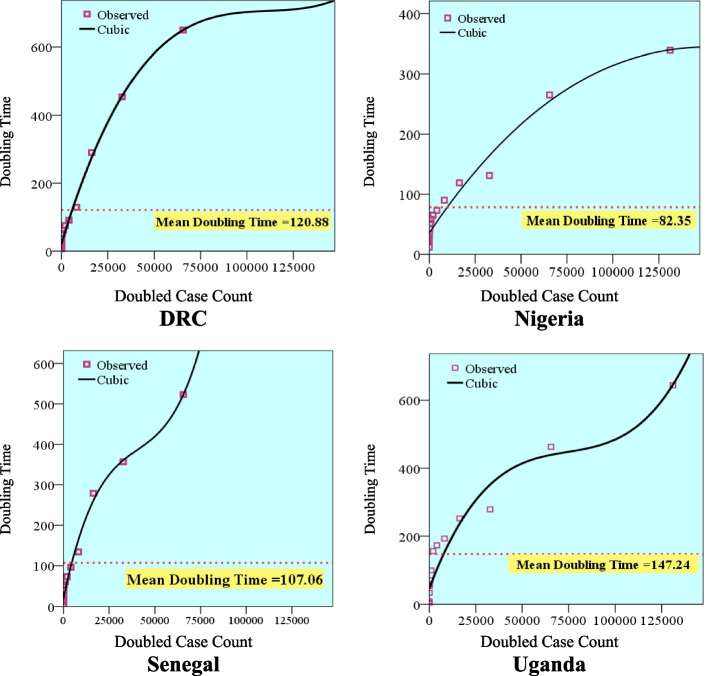
Distribution of Doubling Time against Doubled Case Counts in DRC, Nigeria, Senegal and Uganda

The data in Fig. 3 depict that the average doubling time in COVID-19 case count was 148 days in Uganda, 129 days in the Democratic Republic of Congo, 107 days in Senegal, and 83 days in Nigeria. The cubic model fits perfectly well with the doubling time observed for daily confirmed cases in all the countries with their coefficient of determination (R^2^) being 99.4%, 99.0, 96.7%, and 94.2% in DRC, Senegal, Nigeria, and Uganda respectively. This implies that in DRC for instance, 99.4% of the variation in the data can be attributed to double case count while 0.6% is attributed to other factors (Table 2).

**Table 2:** Model summary and parameter estimates for the fitted cubic model for confirmed COVID-19 cases in DR, Senegal, Nigeria, and Uganda

**Country**	**Model Summary**					**Parameter Estimates**			
	***R***^**2**^	***F***	**df1**	**df2**	**Sig**	**β**_**0**_	**β**_**1**_	**β**_**2**_	**β**_**3**_
**Nigeria**	0.967	126.590	3	13	0.000	35.641	0.005	-1.986E-008	2.160E-014
**Senegal**	0.990	408.845	3	12	0.000	20.301	0.021	-4.387E-007	3.618E-012
**Uganda**	0.942	70.479	3	13	0.000	46.880	0.014	-1.808E-007	8.097E-013
**DRC**	0.994	639.085	3	12	0.000	20.855	0.018	-1.600E-007	4.797E-013

*df* Degree of freedom

**Fig. 4:**
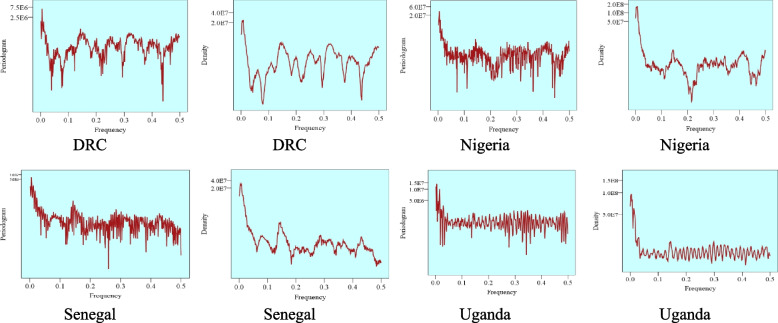
Periodogram and spectra analysis of COVID-19 data in DRC, Nigeria, Senegal, and Uganda

**Fig. 5:**
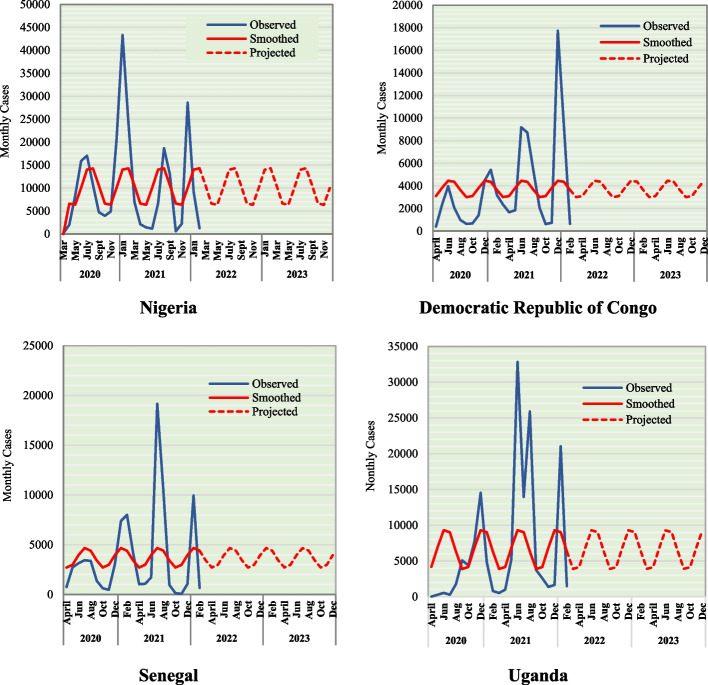
Trend and Trigonometrical modeling of COVID-19 data in CDR, Nigeria, Senegal, and Uganda

In Fig. 4, the periodogram was used to identify the dominant cyclical periods in the COVID-19 data and the possible frequency values that might explain the oscillation pattern of the observed data. The dominant frequencies were used to describe the important periodicities in the series. Parameter estimates for the fitted trigonometric model for confirmed COVID-19 cases are displayed in Table 3. The trend and trigonometrical modeling of COVID-19 data in CDR, Nigeria, Senegal, and Uganda are shown in Fig. 5.

**Table 3:** Parameter estimates for the fitted trigonometric model for confirmed COVID-19 cases in DR, Senegal, Nigeria, and Uganda

**Country**	**Parameters in the Equation**						
	**μ**	**b**_**1**_	**b**_**2**_	**a**	**ω**	**θ**	**k**
**Nigeria**	10,326.08	2328.6398	-3767.837	4429.352	0.5233	-1.61884	3.5
**DRC**	3729.87	95.74851	-793.029	798.7883	0.5233	-1.45064	-0.5
**Senegal**	3691.261	-242.9	979.3778	1009.05	0.5233	-0.24311	3.5
**Uganda**	6594.82	-2726.92	1202.406	2980.249	0.5233	-1.1555	-0.8

In Table 4, the data presented therein are the quarterly number of projected COVID-19 cases and the trend obtained through the moving average method. The projected values assume that the current state of COVID-19 as of the 12^th^ of March, 2022 is sustained throughout the projection period (13^th^ March, 2022 to 31^st^ December, 2023). The seasonal variation was obtained as a difference between the projected and trend values.

**Table 4:** Monthly cases and Trend line of COVID-19 cases in CDR, Nigeria, Senegal, and Uganda

**Year**	**Qtr**	**Nigeria**		**CDR**		**Senegal**		**Uganda**	
		Projected	Trend	Projected	Trend	Projected	Trend	Projected	Trend
2020	Q1								
	Q2	23,083	-	11,377	-	9667	-	20,381	-
	Q3	38,881	28,344	11,005	11,251.26	12,483	10,604	19,199	19,980
	Q4	23,068	33,614.67	11,372	11,128.9	9662	11,544.33	20,360	19,592.67
2021	Q1	38,895	28,339	11,010	11,249.37	12,488	10,602	19,219	19,972.67
	Q2	23,054	33,619.67	11,366	11,130.79	9656	11,546	20,339	19,599.67
	Q3	38,910	28,334.67	11,016	11,247.49	12,494	10,600.67	19,241	19,966
	Q4	23,040	33,624.67	11,360	11,132.68	9652	11,548	20,318	19,607
2022	Q1	38,924	28,330	11,022	11,245.6	12,498	10,598.67	19,262	19,959
	Q2	23,026	33,629.33	11,355	11,134.57	9646	11,549.33	20,297	19,614
	Q3	38,938	28,325.33	11,027	11,243.71	12,504	10,597.33	19,283	19,951.33
	Q4	23,012	33,634	11,349	11,136.46	9642	11,551.33	20,274	19,620.33
2023	Q1	38,952	28,320.67	11,033	11,241.82	12,508	10,595.33	19,304	19,943.67
	Q2	22,998	33,638.67	11,343	11,138.35	9636	11,552.67	20,253	19,627.67
	Q3	38,966	28,316	11,039	11,239.92	12,514	10,594	19,326	19,937
	Q4	22,984	-	11,338	-	9632	-	20,232	-

The data as presented in Table 5 shows the adjusted seasonal variation index (SVI) of COVID-19 in the studied area. This was done with the view to ascertaining what the exact variation should be in each quarter of the year. The data show higher seasonal variation in the first (SVI_Nigeria_ = 10,593.81 & SVI_Senegal_ = 1899.368) and third (SVI_Nigeria_ = 10,593.78 & SVI_Senegal_ = 1899.785) quarters in Nigeria and Senegal than the second (SVI_Nigeria_ = -10,603.2 & SVI_Senegal_ = -1903.3) and fourth (SVI_Nigeria_ = -10,584.4 & SVI_Senegal_ = -1895.85) quarters. The reverse pattern to what was found in Nigeria and Senegal was found in DRC and Uganda where higher seasonal variation was observed in the second and fourth quarters. The pattern found in Nigeria and Senegal shows that more cases of COVID-19 are expected in the first (January – March) and third quarters (July –September) of a year whereas, in DRC and Uganda, more people infected with COVID-19 are likely to be reported in the second (April–June) and fourth (October–December) quarters.

**Table 5:** Estimated seasonal variation adjustment per quarter for number of COVID-19 cases in DRC, Nigeria, Senegal, and Uganda

**Country**	**Year**	**Quarter**				Adjustment
		**1**	**2**	**3**	**4**	Factor
**Nigeria**	1			10,537	-10,547	
	2	10,556	-10,566	10,575	-10,585	
	3	10,594	-10,603	10,613	-10,622	
	4	10,631	-10,641	10,650		
	Average	10,593.78	-10,603.2	10,593.75	-10,584.4	-0.03472
		0.034722	0.034722	0.034722	0.034722	
	Adjusted Seasonal Index	**10,593.81**	**-10,603.2**	**10,593.78**	**-10,584.4**	
**DRC**	1	-	-	-247	243	
	2	-239	235	-232	228	
	3	-224	220	-216	213	
	4	-209	205	-201	-	
	Average	-223.94	220.1616	-223.937	227.7188	0.000618
		-0.00062	-0.00062	-0.00062	-0.00062	
	Adjusted Seasonal Index	**-223.941**	**220.1609**	**-223.938**	**227.7182**	
**Senegal**	1	-	-	1879	-1882	
	2	1886	-1890	1893	-1896	
	3	1899	-1903	1907	-1909	
	4	1913	-1917	1920	-	
	Average	1899.333	-1903.33	1899.75	-1895.89	-0.03472
		0.034722	0.034722	0.034722	0.034722	
	Adjusted Seasonal Index	**1899.368**	***-1903.3***	**1899.785**	**-1895.85**	
**Uganda**	1			-781	767	
	2	-754	739	-725	711	
	3	-697	683	-668	654	
	4	-640	625	-611		
	Average	-696.778	682.5556	-696.333	710.6667	0.027778
		-0.02778	-0.02778	-0.02778	-0.02778	
	Adjusted Seasonal Index	**-696.806**	**682.5278**	**-696.361**	**710.6389**	

## Discussion

In this study, the trajectory of the daily confirmed cases of COVID-19 was assessed to ascertain whether there is seasonality in the occurrence of the disease or not. The trigonometric model, a time series model, was fitted using estimated parameters to project 3-year COVID–19-related data. The seasonality of the data for Nigeria, Uganda, DRC, and Senegal was assessed afterward. We found that the rate of spread of COVID-19 was highest in Nigeria followed by Uganda and Senegal while the lowest rate was observed in DRC. This pattern of the rate of spread is expected since Nigeria and Uganda are more densely populated than DRC and Senegal. The spread of infectious diseases like COVID-19 can be accelerated by large population size [25, 26]. Densely populated areas are characterized by overcrowding, close contact, and poor living conditions including poor sanitation, which serve as breeding grounds for infectious agents and therefore, have the tendency to facilitate disease transmission. The response approaches used in mitigating the spread of the disease in these countries could also be responsible for the observed patterns.

The study revealed that the COVID-19 related deaths per one million population was highest in Senegal, followed by Uganda, Nigeria, and the Democratic Republic of Congo. The Case Fatality Rate (CFR) deviates slightly from this pattern with the highest observed in Senegal, Uganda, DRC, and Nigeria in that order. Despite the demographic and health system similarities among these countries, the differences in political architecture, health workforce, response to the COVID-19 pandemic, and compliance with the public health measures could explain the variation in the observed COVID-19 death patterns among the countries [27–29]. There is also the possibility that factors such as an aging population, poor dietary intake, pre-existing comorbidities like hypertension, ischemic heart disease, smoking, history of cancer, chronic liver disease, and obesity might be more prevalent in countries where higher COVID-19-related deaths per 1,000 population or CFR have been reported. Earlier studies have established that COVID-19 patients with these attributes have higher risks of dying than those where such are absent [30, 31].

Epidemic doubling times depict the sequence of intervals at which the cumulative incidence of the disease doubles. A longer doubling time indicates a slowdown in the COVID-19 disease propagation if the underlying reporting rate remains unchanged [10]. The assessment of the growth rate of COVID-19 cases at the early stage of the disease may not be perfect mainly due to poor testing and difficulties in establishing the cases. However, more accurate figures were obtained over time as better mechanisms and protocols were designed for testing and data streaming. The average doubling time (ADT) for COVID-19 cases in this study reveals a striking divergence in patterns of change across countries. The ADT was highest in Uganda followed by DRC, Senegal, and then Nigeria. Interaction within the population, especially in the absence of vaccines during the first wave of the disease, and differences in the preventive and control measures might explain the observed variation in doubling time across the studied countries. Aside from the general systemic causes like biological characteristics of the virus and incubation period that are the same for all societies, other factors which might contribute to the variation in ADT of COVID-19 across the analyzed countries included differences in the; age structure, sex composition, immune system, poor hygienic practice, air pollution, meteorological conditions, daily inter-personal contacts, and societal context [32]. These factors are important predictors of diffusion of viral infectivity [32].

Seasonal patterns in infectious disease occurrence are common in both temperate and tropical climates. Factors like human activity, variability in human immune system function, variations in vitamin D levels, seasonality of melatonin, and pathogen infectivity have been flagged and proposed to explain the seasonality of infectious diseases [10, 33, 34]. For instance, Vitamin D supplementation tends to reduce the incidence of acute respiratory infection by controlling the expression of specific endogenous antimicrobial peptides in immune cells, modulating the immune response, and the course of autoimmune processes [35]. It prevents loss of neural sensation in COVID-19 by stimulating the expression of neurotrophins like Nerve Growth Factor (NGF): Vitamin D: Induction of key neurotrophic factors [36]. The hormone melatonin is effective in combating various bacterial and viral infections. It can influence seasonal changes in immune function observed in humans through changes in the duration of melatonin secretion which often account for the seasonal pattern of symptom expression shown by infectious diseases [10]. Generally, the coronavirus is a seasonal virus. These data showed that more cases of COVID-19 are expected in the first (January – March) and third quarters (July –September) of a year in Nigeria and Senegal whereas, in DRC and Uganda, more cases of COVID-19 may likely be reported in the second (April – June) and fourth (October–December) quarters. The pattern of seasonality demonstrated by the data is interesting in that the countries that shared the same pattern are from the same regional blocks in Africa—while Nigeria and Senegal are from western Africa, the DRC and Uganda are two neighboring countries in Central and East Africa. These within-region similarities may be due to resemblances in response, sociocultural and ecological factors. In Nigeria and Senegal, the first quarter of each year is characterized by cold weather, the third quarter is often the peak of the rainy season and is known for cultural celebrations which possibly could facilitate higher COVID-19 spread than the second and last quarter of the year.

The episode of the COVID-19 pandemic is not up to 3 years, therefore examination of seasonality in the data based on 2-year data might face the challenge of robustness. However, the use of a trigonometric model to predict for an additional year before the seasonality’s assessment adjusted for this challenge. It is therefore pertinent to note that the observed third-year data might deviate from the assumption-based generated data predicted through the use of the trigonometric model. Especially, if the constellation of causal and preventive factors like vaccination and restrictive measures are different from the current situation in the third year. The assumption that all things being equal (ceteris paribus) i.e. if the current situation is sustained over the projected period might not be realistic at that time. Therefore, the readers must be cautious of this fact while interpreting the findings. The analyzed data relied on the COVID-19 database of an institution which is often updated on daily bases, this may not fully reflect the exact number of COVID-19 cases captured within the country. The inadequate state of data from the disease surveillance system peculiar to less developed countries cannot be ignored from the analyzed COVID-19 data. Therefore, there is the possibility of some minor errors in the data which we envisaged that the use of the trigonometric model has resolved.

## Conclusion

The COVID-19 spread in Senegal, Nigeria, DRC, and Uganda followed an increasing trend and exhibited seasonality in the pattern of spread with a short doubling time. There was also substantial variation in the timing of the cases of COVID-19 between countries. More cases were reported in the first and third quarters of the year in Nigeria and Senegal and the second and third quarters of the year in Uganda and the Democratic Republic of Congo. These variations emphasize the need to target particular seasons of the year for COVID-19 preparedness and response action.

## Data Availability

The datasets zanalyzed during the current study are available in the [Worldometer; European Centre for Disease Prevention and Control] repository, (https://www.worldometers.info/coronavirus/; https://www.ecdc.europa.eu/en/publications-data/surveillance-systems-overview-2021).

## Abbreviations

ADT: Average Doubling Time
CFR: Case Fatality Rate
SVI: Adjusted Seasonal Variation Index
COVID-19: Coronavirus disease

## References

[1] Worldometers.info. Worldometer. Dover; 2020. https://www.worldometers.coronavirus/.

[2] European Centre for Disease Prevention and Control. An agency of the European Union. https://www.ecdc.europa.eu/en/publications-data/surveillance-systems-overview-2021.

[3] Schellekens P, Sourrouille D (2020). COVID-19 mortality in rich and poor countries: a tale of two pandemics? Policy research working paper; no. 9260.

[4] Osei SA, Biney RP, Anning AS, Nortey LN, Ghartey-Kwansah G (2022). Low incidence of COVID-19 case severity and mortality in Africa; could malaria co-infection provide the missing link?. BMC Infect Dis.

[5] Bayati M (2021). Why is COVID-19 more concentrated in countries with high economic status?. Iran J Public Health.

[6] Aloke C, Ganesh B, Obasi NA, Aja PM, Ugwuja EI, Nwankwo JO (2022). An overview of COVID-19 in sub-Saharan Africa: the transmissibility, pathogenicity, morbidity and mortality so far. Jordan J Biol Sci.

[7] Rigdon SE, Turabelidze G, Jahanpour E. Trigonometric regression for analysis of public health surveillance data. 2014. 10.1155/2014/673293.

[8] Siettos CI, Russo L (2013). Mathematical modeling of infectious disease dynamics. Virulence.

[9] Spelman T, Gray O, Lucas R, Butzkueven H. A method of trigonometric modelling of seasonal variation demonstrated with multiple sclerosis relapse data. J Vis Exp. 2015;(106):e53169. 10.3791/53169. 10.3791/53169 PMC4692792 26709960

[10] Fares A (2013). Factors influencing the seasonal patterns of infectious diseases. Int J Prev Med.

[11] Poole L. Seasonal influences on the spread of SARS-CoV-2 (COVID19), causality, and forecastabililty (3-15-2020) (March 15, 2020). 10.2139/ssrn.3554746.

[12] Hoogeveen MJ, Van Gorp ECM, Hoogeveen EK (2021). Can pollen explain the seasonality of flu-like illnesses in the Netherlands? Sci. Total Environ.

[13] D’Amico F, Marmiere M, Righetti B, Scquizzato T, Zangrillo A, Puglisi R, Landoni G (2022). COVID-19 seasonality in temperate countries. Environ Res.

[14] Shuaib F, Gunnala R, Musa EO, Mahoney FJ, Oguntimehin O, Nguku PM (2014). Ebola virus disease outbreak—Nigeria, July–September 2014. MMWR Morb Mortal Wkly Rep.

[15] IlungaKalenga O, Moeti M, Sparrow A, Nguyen V-K, Lucey D, Ghebreyesus TA (2019). The ongoing Ebola epidemic in the Democratic Republic of Congo, 2018–2019. N Engl J Med.

[16] Mirkovic K, Thwing J, Diack PA (2014). Importation and containment of Ebola virus disease—Senegal, August–September 2014. MMWR Morb Mortal Wkly Rep.

[17] Lamunu M, Lutwama J, Kamugisha J, Opio A, Nambooze J, Ndayimirije N (2004). Containing a haemorrhagic fever epidemic: the Ebola experience in Uganda (October 2000–January 2001). Int J Infect Dis.

[18] United Nations, Department of Economic and Social Affairs, Population Division. World Population Prospects (2019). custom data acquired via website.

[19] World Population Review. Tropical countries 2023. https://worldpopulationreview.com/country-rankings/tropical-countries. Accessed 2 Aug 2023.

[20] World Population Review. Countries by population density 2023. https://worldpopulationreview.com/. Accessed 17 Sep 2023.

[21] Oyugi B. How African countries coordinated the response to COVID-19: lessons for public health. The CONVERSATION; 2021. https://theconversation.com/how-african-countries-coordinated-the-response-to-covid-19-lessons-for-public-health-187299.

[22] Oleribe OO, Suliman AAA, Taylor-Robinson SD, Corrah T (2021). Possible reasons why sub-Saharan Africa experienced a less severe COVID-19 pandemic in 2020. J Multidiscip Healthc.

[23] Essential Health Services: Nigeria. Exemplars in global health. 2022. Stephen N. Kabwama, Suzanne N. Kiwanuka, Olufunmilayo I Fawole, David M. Dairo, Ayo S. Adebowale, Segun Bello, Eniola A Bamgboye, Rotimi F. Afolabi, Mobolaji M. Salawu, Rhoda K. Wanyenze Available: https://www.exemplars.health/emerging-topics/epidemic-preparedness-and-response/essential-health-services/nigeria. Accessed 30 Apr 2023.

[24] Shumway RH, Stoffer DS. Time series analysis and its applications. 2017. ISBN: 978-3-319-52451-1. https://link.springer.com/book/10.1007/978-3-319-52452-8

[25] Ganasegeran K, Jamil MFA, Ch’ng ASH, Looi I, Peariasamy KM (2021). Influence of population density for COVID-19 spread in Malaysia: an ecological study. Int J Environ Res Public Health.

[26] Tarwater PM. The effects of population density on the spread of disease. Texas Medical Center Dissertations (via ProQuest); 1999. https://digitalcommons.library.

[27] James A, Dalal J, Kousi T, Vivacqua D, Câmara DCP, Dos Reis IC, Botero Mesa S, Ng’ambi W, Ansobi P, Bianchi LM, Lee TM, Ogundiran O, Stoll B, Chimbetete C, Mboussou F, Impouma B, Hofer CB, Coelho FC, Keiser O, Abbate JL (2022). An in-depth statistical analysis of the COVID-19 pandemic’s initial spread in the WHO African region. BMJ Glob Health.

[28] Tsinda EK, Mmbando GS (2021). Recent updates on the possible reasons for the low incidence and morbidity of COVID-19 cases in Africa. Bull Natl Res Cent.

[29] Okonji EF, Okonji OC, Mukumbang FC, Van Wyk B (2021). Understanding varying COVID-19 mortality rates reported in Africa compared to Europe, Americas and Asia. Trop Med Int Health.

[30] Elezkurtaj S, Greuel S, Ihlow J (2021). Causes of death and comorbidities in hospitalized patients with COVID-19. Sci Rep.

[31] Bhaskaran K, Bacon S, Evans SJ, Bates CJ (2021). Factors associated with deaths due to COVID-19 versus other causes: population-based cohort analysis of UK primary care data and linked national death registrations within the OpenSAFELY platform. Lancet Reg Health Eur.

[32] Coccia M (2020). Factors determining the diffusion of COVID-19 and suggested strategy to prevent future accelerated viral infectivity similar to COVID. Sci Total Environ.

[33] Vynnycky E, White RG (2010). An introduction to infectious disease modelling.

[34] Grassly NC, Fraser C (2006). Seasonal infectious disease epidemiology. Proc Biol Sci.

[35] Pigarova EA, Povalyaeva AA, Dzeranova LK, Rozhinskaya LY, Mokrysheva NG (2020). The role of vitamin D in seasonal acute respiratory viral infections and COVID-19. Ter Arkh.

[36] Xu Y, Baylink DJ, Chen CS, Reeves ME, Xiao J, Lacy C, Lau E, Cao H (2020). The importance of vitamin d metabolism as a potential prophylactic, immunoregulatory and neuroprotective treatment for COVID-19. J Transl Med.

